# Editorial: Insights in healthcare professions education: 2023

**DOI:** 10.3389/fmed.2024.1438116

**Published:** 2024-07-09

**Authors:** Lynn V. Monrouxe, Jacqueline Bloomfield

**Affiliations:** Faculty of Medicine and Health, The University of Sydney, Camperdown, NSW, Australia

**Keywords:** dignity, telehealth, curriculum evaluation models, pragmatism, wellbeing, simulation teaching method

## Introduction

Welcome to our collection of articles in the 2023 edition of the Research Topic “*Insights in healthcare professions education*” where we explore the latest developments in education, research methodologies and evaluation approaches that are shaping the future of medical and healthcare education. In this Research Topic of fifteen articles from across the world, we identify three key themes arising from this contemporary body of research (1) Course and Program Evaluation and development; (2) Innovative Teaching Approaches and Technology Integration; and (3) Promoting Human-Centered Education and Practice. Indeed, as healthcare landscapes continue to evolve for us all, educators across the world face the challenge of equipping students with the skills and knowledge necessary for practice while emphasizing ethical decision-making, diversity, and growth. By exploring the articles within these three identified themes, we believe you will gain a comprehensive view of the transformative impact of educational research on the next generation of healthcare professionals.

## Course and program evaluation and development

Five articles in our Research Topic focus on evaluating and improving healthcare education interventions and programs across a range of healthcare professional groups, using different frameworks, tools and methodologies to assess course quality, acceptability of service learning, validity of assessment and guide curriculum design (Choi et al.; Guraya et al.; Li et al.; van de Mortel et al.; Van Winkle et al.).

Li et al., employing the well-established approach of Generalizability theory (G-theory), examined the correlation between items of their core residency entrustable professional activities. In their paper they outline their rationale for G-theory, further embedding their work in the rigor required for our developing field. Carefully following all steps required for G-theory, they concluded that both residents' and trainers' ratings showed good validity and reliability in the pediatric residency training program at the Peking University First Hospital. Using a different approach and focusing on students attitudes to learning specific courses, Van Winkle et al. report on the development of a new measurement instrument designed to assess prospective medical students' attitudes toward team service-learning in an Immunology course. Analyzing remote and in-person students' data (*n* = 73) from a 10-item survey measuring attitudes toward team-service learning, community service and teammates, they identified a single factor of “attitudes toward required service-learning”. Although further testing of their tool's reliability is needed, especially with other healthcare professions students and across different cultural contexts, we warmly welcome this new assessment tool that has the potential to detect differences in attitudes toward service-learning across different groups of students and different contexts, enabling course developers to implement curricula changes when needed.

Two studies in our Research Topic offer very different approaches to understanding and improving curricula quality. Underpinned by Social Cognitive Theory, van de Mortel et al. advocate for the implementation of an holistic, collaborative, and systematic Program Quality Framework. Choi et al., however, using a pragmatic approach, employ the six-step “contribution analysis”. Each approach has its merits with the Program Quality Framework fostering the development of staff capability and scholarship throughout the curriculum's duration with educational scholarship underpinning curricula design and teaching. Choi et al.'s contribution analysis moves away from measurements of students' attitudes, toward an appreciation of the deeper complexity around educational offerings and uptake. Indeed, rather than offering a single measurement of value, contribution analysis provides an holistic understanding of the multifaceted trajectories experienced by students as they progress through their learning journey.

Interestingly, the research perspective of pragmatism features heavily across two articles (Choi et al.; Guraya et al.). While taking us carefully through the 6-step contribution analysis process, Choi et al., explain how they examined the factors involved in students' development of their graduate outcomes. They also report on their evidence-based model illustrating the relationships between external factors, graduate outcomes, results, assumptions and risks. Similarly, Guraya et al. carefully outline their philosophical approach of pragmatism, alongside the materials, methods and data analysis they adopted whilst developing, validating and evaluating their e-professionalism framework in the context of medical education. Interestingly, unlike Choi et al., who examine the complexity of both content and processes within education, and Van Winkle et al. who focus on students' attitudes, Guraya et al. employ the theory of planned behavior and Kirkpatrick's model of evaluation to focus on outcomes at the behavioral level and how their framework has impacted on how learners engage in the digital world. Furthermore, they describe in impressive detail the great lengths they have taken to conceive of, implement and evaluate their e-professionalism framework; a level of detail that is often missing or unspoken.

## Innovative teaching approaches and technology integration

Four articles in our Research Topic focus on the use of the technologies within healthcare education. These include: simulation, virtual/augmented reality, telehealth, interactive online experiences, and social media with the studies exploring when and how to integrate them effectively into curriculum (Attar et al.; Bacon et al.; Lembo et al.; Stoehr et al.). For example, although Bacon et al., found face-to-face consultations to be significantly more acceptable, affording more opportunities for students than telehealth, telehealth has its place in the curriculum, providing valuable learning experiences for students and supporting their competency development. Notably, in this study, students reported improvements in clinical reasoning, problem-solving skills, resourcefulness, flexibility, and communication skills in ways that are quite different to their face-to-face experience. Furthermore, students' skills development (e.g., in areas such as craniotomies, dural openings and suturing) has shown to be enhanced by learning with devices such as Google Glass for Augmented Reality and a cadaver-free high-fidelity simulator (Lembo et al.). In this pilot study of neurosurgery residents' and students' learning during a 6-week course, all participants significantly improved across technical indicators, with more junior participants showing greater improvement.

Stoehr et al., report on their blended approach to medical students' thoracic radiology learning using both online and onsite learning. Interactive online learning included on-demand video loops, image magnification and clickable color highlighting whereas the in-person teaching comprised small-group (8–10) 90-min seminars. Significant improvements in knowledge were found, with the online aspect of the course motivating students to become more involved with thoracic radiology in the future. Finally, focusing on phlebotomists (*n* = 435) from across 14 public hospitals in the Eastern region of Saudi Arabia, Attar et al. examined their use of social media to improve their knowledge and awareness of safety procedures and practices. They concluded that social media is a valid form of knowledge exchange and development with over 70% of participants reporting that that they frequently shared phlebotomy-related information with other phlebotomists. With this in mind, Attar et al. highlight that the need to now focus on improving skills, knowledge and awareness regarding identifying misinformation to prevent inadvertently sharing incorrect knowledge among colleagues.

## Promoting human-centered education and practice

Four articles in our Research Topic focus on the importance of placing human values and dignity at the forefront of healthcare education and practice (Fu et al.; González-Blázquez et al.; Klinner et al.; Schramlová et al.). They encompass the ethical considerations of providing educational opportunities that respect the dignity of all individuals, ensuring equality in healthcare, prioritizing student wellbeing and enhancing their quality of life throughout their educational journey. We would further argue that ethics training in healthcare plays a vital role in promoting dignity, student wellbeing and quality of life, healthcare equality (including LGBTQ+) by embedding ethical principles into education and practice, thereby fostering a culture of respect, justice, and moral integrity. However, González-Blázquez et al., in their systematic review of healthcare students' bioethical knowledge and training found that healthcare students, generally, lack sufficient knowledge and/or skills to resolve the ethical dilemmas they may encounter during clinical practice, although the extent of this differed among healthcare discipline and country of study.

Dilemmas around dignity during workplace learning is the focus of another study. Klinner et al. examined a range of stakeholders' understanding of what comprises dignity in workplace learning in healthcare. Interviewing students, placement educators and university staff across seven allied health professions groups (*n* = 51), they identified eight distinct dimensions of what comprises dignity in a service-learning environment, including: feeling safe, respected, equal, having a sense of belonging as well as the relationship we have with ourselves (e.g., self-worth, self-respect, self-compassion, and self-understanding). Related to the issue of dignity in the workplace, Fu et al. examined the knowledge and attitudes of medical students from Singapore and United Kingdom (UK) toward lesbian, gay, bisexual, transgender, queer, and other sexual and gender minority (LGBTQ+) individuals. Amongst their findings is that, despite espousing positive attitudes, participants lacked a general understanding of commonly used vocabulary and topics, and this was more pronounced for Singaporean participants. Furthermore, 84% of students had no LGBTQ+ specific training in their university education emphasizing the need for inclusive curricula to achieve a dignified experience for all.

The final article we highlight in this Research Topic theme links with Klinner et al.'s finding around dignity being related to the relationship we have with ourselves, specifically to self-care, as it focuses on the wellbeing and quality of life of students in bachelor's physiotherapy programs (*n* = 1,075) across 23 European faculties, in eight countries (Schramlová et al.). Issues such as mental wellbeing, stress levels, sleep quality, and adherence to physical activity and nutritional guidelines were examined within the broader framework of *quality of life*. Schramlová et al. found (amongst other things) that academic factors (e.g., amount of material to learn, exam frequency) were strongly related to higher stress levels, with women being more vulnerable than men. Furthermore, problems with sleep, nutrition and physical activity were also reported.

## Further studies

Notably, two further studies included in this Research Topic sit outside these themes (Alhamami; Jain et al.). Reporting a systematic review of the evolution and impact of English as a Medium of Instruction (EMI) in healthcare education over the past decade, Alhamami presents a distinctive opportunity for us to examine how EMI has affected students, educational institutions, and the broader landscape of healthcare education. Further, Jain et al. reports on Latvia's medical education system and its challenges, including institutional capacity constraints and investments in adapting to changing landscapes. In doing so, this interesting report and provides us with a unique insight into medical education in Latvia and an opportunity to identify differences and similarities between this and that within our own country.

## Conclusion

In conclusion, the diverse array of articles presented in our latest Research Topic underscores the multifaceted nature of healthcare education. By evaluating and enhancing educational interventions across various professional groups, exploring the integration of cutting-edge technologies, and prioritizing humanistic values essential to healthcare, these studies collectively advance our understanding of effective healthcare education. The insights gained from this compilation not only highlight the importance of rigorous assessment and innovative teaching methods but also emphasize the ethical imperative of fostering environments that uphold dignity, equality, and wellbeing. As we move forward, these contributions will be instrumental in shaping healthcare curricula that produce competent, compassionate, and well-rounded healthcare professionals across the world.

## Author contributions

LM: Writing – original draft, Writing – review & editing. JB: Writing – review & editing.

